# Diminished TLR2-TLR9 mediated CD4+ T cell responses are associated with increased inflammation in intraocular tuberculosis

**DOI:** 10.1038/s41598-018-32234-3

**Published:** 2018-09-14

**Authors:** Ravi Kumar Sharma, Jyoti Sharma, Zafar K. Khan, Ajinkya Pattekar, Vishali Gupta, Reema Bansal, Kusum Sharma, Ashutosh Nath Aggarwal, Amod Gupta, Naresh Sachdeva

**Affiliations:** 1 Advanced Eye Centre, Post Graduate Institute of Medical Education and Research, Chandigarh, India; 2 Department of Microbiology and Immunology and the Institute for Molecular Medicine and Infectious Disease, Drexel University College of Medicine, Philadelphia, PA USA; 3 Department of Medical Microbiology, Post Graduate Institute of Medical Education and Research, Chandigarh, India; 4 Department of Pulmonary Medicine, Post Graduate Institute of Medical Education and Research, Chandigarh, India; 5 Department of Endocrinology, Post Graduate Institute of Medical Education and Research, Chandigarh, India

## Abstract

Intraocular tuberculosis (IOTB) is amongst the leading causes of uveitis in tropical countries. Despite reports on involvement of proinflammatory cytokines, studies on innate immune responses in disease pathogenesis are lacking. Reports from animal models and patients with pulmonary tuberculosis indicate that defects in toll like receptor (TLR)2 and TLR9 signalling predispose them to tuberculosis. In this context, we investigated the role of TLR2, TLR4 and TLR9 in generation of CD4+ T effector (Teff) cell responses during IOTB. Firstly, the cells in vitreous fluids showed lower expression of TLR2 and TLR9 in IOTB as compared to non-uveitis and non-TB uveitis groups. Next, peripheral CD4+ Teff cells of subjects with IOTB showed decreased proliferative responses and lower induction of Tregs following TLR2 and TLR9 stimulation. Further, TLR9 ligation resulted in increased IFN-γ and IL-17a but decreased expression of IL-10 and TGF-β. Lastly, lower expression of genes involved in TLR9 signalling after direct TLR9 ligation was observed in IOTB. Collectively, our results show that a subdued response to direct TLR2 and TLR9 stimulation in CD4+ T cells is associated with increased proinflammatory responses in IOTB. These findings reveal an important link between innate immune signalling and ensuing adaptive immune responses in IOTB with implications in other forms of extrapulmonary tuberculosis.

## Introduction

Intraocular tuberculosis (IOTB) or tubercular uveitis is one of the leading causes of uveitis in tropical countries, including India and China^1,2^. The guidelines on diagnosis, classification and management of the disease have already been reported by our group^3–6^, including the detection of mycobacterial DNA, a key evidence of mycobacterial involvement, in vitreous fluids of patients with IOTB^6,7^. Isolated reports on immune responses in IOTB have suggested higher levels of inflammatory cytokines, IFN-γ, IL-6, IL-8 along with T cell chemoattractants in aqueous humor of subjects with IOTB^8,9^. We have also reported enhanced levels of proinflammatory cytokines, IFN-γ and IL-17A in vitreous humor of patients with IOTB, accompanied with lower frequency of CD4+ regulatory T cells (Tregs) in their peripheral blood^10^. However, the roles of active *Mycobacterium tuberculosis* infection in disease initiation and subsequent host responses are still unclear, making the studies involving innate immune factors a prerequisite for better understanding of pathology of IOTB.

The primary responders in innate immune response are toll like receptors (TLRs) that are highly expressed on antigen presenting cells (APCs), such as dendritic cells and macrophages. TLRs recognize conserved molecular patterns, pathogen associated molecular patterns and modulation of immune responses by TLRs can have significant impact on the resulting adaptive immune responses. In experimental models of other forms of uveitis, such as endotoxin induced uveitis (EIU), it has been found that ocular inflammation results simply via endotoxin mediated activation of innate immune system^11^. In IOTB, where there is still ambiguity on the immunogenic entity, an insight on the role of TLRs becomes important. Here, the only indicative evidence of the presence of a foreign TLR ligand in the eye is mycobacterial DNA, a TLR9 ligand, as shown by our group and others^6,12^. In this context, we recently observed that T cells form a major proportion of ocular infiltrating cells in IOTB and these infiltrated CD4+ T cells show lower uptake of TLR9 ligand, ODN 2216, than the peripheral CD4+ T cells^13^. Considering these two observations, assessment of CD4+ T cell responses to TLRs, particularly TLR9, in subjects with IOTB can provide insights on exaggerated ocular inflammation observed in these subjects. Interestingly, the studies on experimental models of tuberculosis and patients with primary tuberculosis also indicate that a defect in TLR9 signalling predisposes them to the disease^14,15^.

Besides APC mediated stimulation, direct ligation of TLR ligands has varying effects on adaptive immune cells, particularly Tregs^16–19^. A previous study showed selective expression of TLR4, 5 and 8, and increased suppressive potential in Tregs after TLR4 stimulation^16^. In contrast, TLR2 stimulation showed increased proliferation of Tregs, but decline in suppressive ability^17^. Similarly, ligation of TLR8^18^ and TLR9^19^ was shown to decrease their suppressive ability. In view of these findings, we hypothesise that exposure to a consistently present TLR ligand may further influence the outcome of local immune response in IOTB. Therefore, we investigated the expression of TLR2, TLR4 and TLR9 in vitreous fluids of subjects with IOTB and compared the functional responses of peripheral CD4+ Teff cells towards these TLR stimuli. Further, we assessed the impact of TLR stimulation on induction of Tregs from CD4+ Teff cells in the disease. We provided evidence that IOTB involves a subdued response to TLR2 and TLR9 stimulation and in particular, direct TLR9 signalling in CD4+ Teff cells, which manifests into lower Treg induction and elevated proinflammatory responses. We could further demonstrate association between TLR2 and TLR9 mediated CD4+ Teff cell function and ocular inflammation in IOTB.

## Results

### Subject characteristics

The mean (±SEM) age of subjects with confirmed IOTB^3^, was 42.41 ± 2.52 years. The disease spectrum in IOTB included, pan uveitis (n = 4), vitritis (n = 3), intermediate uveitis (n = 6), subretinal abscess (n = 1) and multifocal choroiditis (n = 4). None of the subjects in IOTB group had any evidence of extraocular tuberculosis or any other manifestation of tuberculosis in other parts of the body. Majority of these subjects had latent tuberculosis as evidenced by a positive mantoux reaction (>10 mm induration after 48 hours) (n = 15/18). The disease spectrum in non-TB uveitis group included pan uveitis (n = 6), vitritis (n = 4), intermediate uveitis (n = 4) and endogenous endophthalmitis (n = 1), with mean age 39.30 ± 5.68 years. Non-uveitis control group included subjects with macular hole (n = 8), dropped intraocular lens (n = 5) and epiretinal membrane (n = 2), with mean age of 48.87 ± 3.42 years. The mean age of subjects with active tuberculosis (n = 12) was 39.29 ± 8.19 years. There was no difference in age distribution among the groups, ruling out the role of age as a contributing factor in immune responses observed.

### TLR2 and TLR9 expression has a negative correlation with ocular inflammation in IOTB

**Figure 1:**
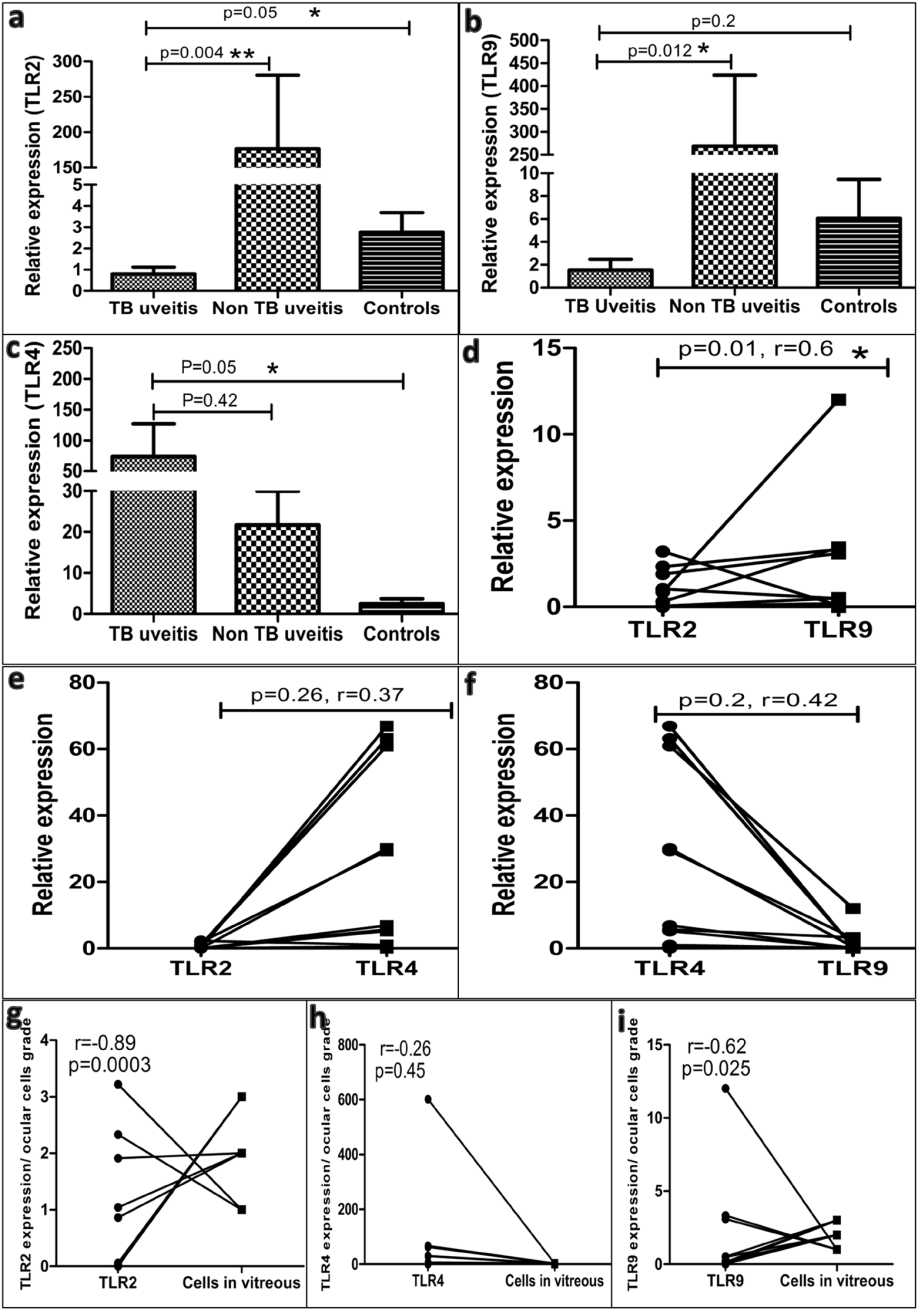
Toll like receptor mRNA levels in vitreous humor. The mRNA levels of TLR2, TLR4 and TLR9 were assessed in cells obtained from vitreous humor of subjects with IOTB by real time RT-PCR. Non-uveitis and non-TB uveitis subjects were used as controls. (**a**) The expression of TLR2 was significantly lower in subjects with IOTB as compared to non-TB uveitis (p = 0.004) and non-uveitis control (p = 0.05) subjects. (**b**) The expression of TLR9 was also lower in subjects with IOTB as compared to non-TB uveitis (p = 0.012), but not non-uveitis control (p = 0.2) subjects. (**c**) In contrast, the expression of TLR4 was significantly higher in subjects with IOTB as compared to non-uveitis controls (p = 0.05) but similar to the non-TB uveitis controls (p = 0.42). (**d**) The co-expression of TLRs was further compared and there was a positive correlation between TLR2 and TLR9 expression in subjects with IOTB (p = 0.01, r = 0.6). However, no significant correlation was observed in expression of (**e**) TLR2-TLR4 (p = 0.26, r = 0.37) and (**f**) TLR4-TLR9 (p = 0.2, r = 0.42) in the disease group. To assess association between TLR expression and ocular inflammation in patients with IOTB, correlation analysis was performed between number of cells in vitreous and (**g**) TLR2 expression, (**h**) TLR4 expression and (**i**) TLR9 expression in vitreous samples. Number of cells in vitreous humor had a negative association with TLR2 (r = −0.89, p = 0.0003) and TLR9 (r = −0.62, p = 0.025) expression but not TLR4 expression (r = −0.26, p = 0.45).

We observed significantly lower mRNA levels of TLR2 in cells of vitreous humor in IOTB subjects (0.8 ± 0.32) as compared to non-TB uveitis (176.6 ± 104.1) (p = 0.004) and non-uveitis subjects (2.77 ± 0.92) (p = 0.05) (Fig. 1a). Interestingly, we observed a similar trend in expression of TLR9, with IOTB (1.55 ± 0.93) showing significantly lower mRNA levels of the receptor as compared to non-TB uveitis (268.5 ± 155.5) (p = 0.01) but not non-uveitis subjects (6.06 ± 3.4) (p = 0.2) (Fig. 1b). However, in contrast to TLR2 and TLR9, the mRNA levels of TLR4 were significantly higher in IOTB (73.72 ± 53.33) as compared to non-uveitis subjects (2.44 ± 1.27) (p = 0.05) (Fig. 1c). Further, mRNA levels of TLR4 were similar between IOTB and non-TB uveitis groups (21.73 ± 8.25) (p = 0.42) (Fig. 1c).

After observing similarity in expression of TLR2 and TLR9, we assessed the correlation between TLR2-TLR4, TLR4-TLR9 and TLR2-TLR9 expression in IOTB subjects. Consistent with the expression levels, we found a significant positive correlation between mRNA levels of TLR2 and TLR9 in IOTB subjects (p = 0.01, r = 0.6) (Fig. 1d). No association was observed in case of TLR2-TLR4 (p = 0.26, r = 0.37) (Fig. 1e) or TLR4-TLR9 (p = 0.2, r = 0.42) (Fig. 1f) expression. The expression of the observed TLRs did not show any correlation in non-TB uveitis or non-uveitis control subjects. Hence, the results clearly showed a diminished TLR2-TLR9 expression in IOTB.

In order to study the relation between TLR expression and inflammation in vitreous humor, we performed correlation analysis between individual TLR expression and qualitative score of number of cells in vitreous, as a measure of ocular inflammation. We observed a negative correlation between TLR2 (r = −0.89, p = 0.0003) (Fig. 1g) and TLR9 (r = −0.62, p = 0.025) (Fig. 1i) expression and the number of cells in vitreous humor in IOTB. However, there was no significant correlation between TLR4 expression and ocular inflammation (r = −0.26, p = 0.45) (Fig. 1h) in IOTB.

### TLR2 and TLR9 ligation results in lower proliferation of Teff cells in IOTB

The effects on proliferation of T cells after direct TLR9 stimulation have been reported in few studies^20–22^. To assess the impact of TLR ligation in IOTB, we compared proliferative responses after exposure with different TLR ligands in Teff cells.

**Figure 2:**
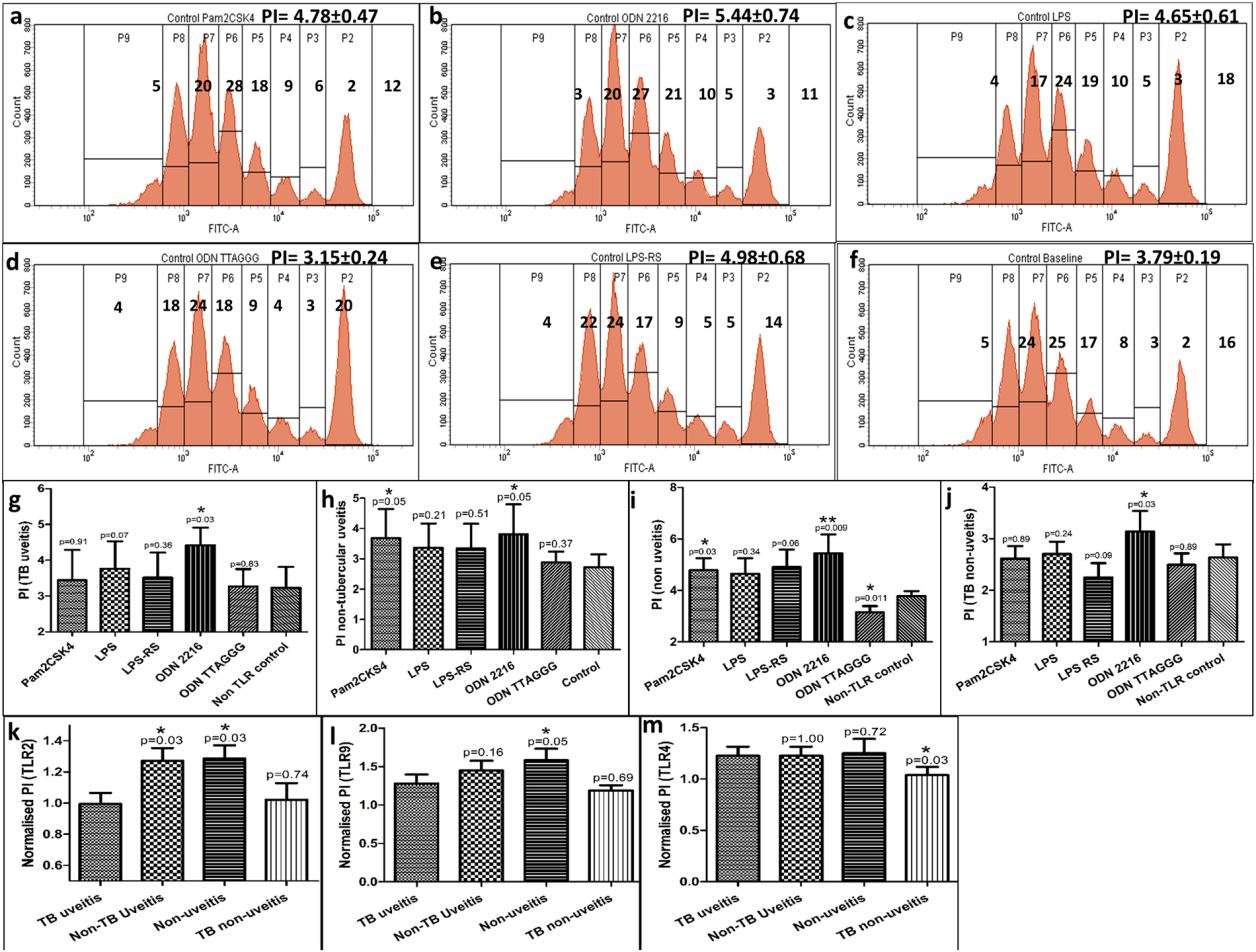
The effect of TLR ligand stimulation on proliferation of CD4+ Teff cells in non-uveitis group. CD4+ Teff cells were stimulated using TLR ligands to assess their impact on cell proliferation. The representative figures show proliferation peaks along with the percentage frequency of dividing and non-dividing cells in a non-uveitis control subject. The stimulations were performed with (**a**) Pam2CSK4, (**b**) ODN 2216, (**c**) LPS, (**d**) ODN TTAGGG, (**e**) LPS-RS, and (**f**) anti CD3/CD28 + IL-2 only (baseline, non-TLR control). (**g**) In IOTB subjects, we observed a significant increase in PI after stimulation with ODN 2216 (p = 0.03) as compared to non-TLR controls. (**h**) In non-TB uveitis controls, there was a significant increase in PI after stimulation with Pam2CSK4 (p = 0.05) and ODN 2216 (p = 0.05) as compared to non-TLR controls. (**i**) In non-uveitis controls, there was a significant increase in PI after stimulation with Pam2CSK4 (p = 0.03) and ODN 2216 (p = 0.009), while significant decrease after ODN TTAGGG treatment (p = 0.011), as compared to non-TLR controls. (**j**) Non-uveitis TB subjects showed a significant increase in PI after stimulation using ODN 2216 (p = 0.03) as compared to non-TLR controls. (**k**) TLR2 stimulation resulted in lower normalized proliferative response in IOTB subjects as compared to non-uveitis (p = 0.03) and non-TB uveitis subjects (p = 0.03). (**l**) IOTB subjects showed a lower proliferative response to ODN 2216 stimulation as compared to non-uveitis subjects (p = 0.05). (**m**) Non-uveitis TB subjects showed lower proliferative response to TLR4 stimulus as compared to other groups.

Firstly, among the non-uveitis control subjects, the stimulation of Teff cells was compared with reference to non-TLR controls. We observed a significant increase in proliferation index (PI) of CD4+ Teff cells after stimulation with TLR2 agonist, Pam2CSK4 (4.78 ± 0.47, p = 0.03) (Fig. 2a,i) and TLR9 agonist, ODN 2216 (5.44 ± 0.74, p = 0.009) (Fig. 2b,i) but not TLR4 agonist LPS (4.65 ± 0.61, p = 0.34) (Fig. 2c,i) as compared to non-TLR controls (3.79 ± 0.19) (Fig. 2f,i). TLR9 antagonist, ODN TTAGGG treatment resulted in a significant decrease in PI than non-TLR controls (3.15 ± 0.24, p = 0.011) (Fig. 2d,i). However, TLR4 antagonist, LPS-RS treatment resulted in similar PI (4.98 ± 0.68, p = 0.06) (Fig. 2e,i). Subsequently, stimulation of Teff cells was compared within IOTB subjects as well as non-TB uveitis and non-uveitis TB control groups. Only ODN 2216 stimulation (4.42 ± 0.49) (Supp. Fig. 1a) resulted in a significant increase in PI than non-TLR controls (3.24 ± 0.58, p = 0.03) (Supp. Fig. 1f) (Fig. 2g) in subjects with IOTB. Other TLR stimuli showed similar proliferation as in non-TLR controls within the IOTB group (Supp. Figs 1b–f and 2g). In non-TB uveitis, Pam2CSK4 (3.69 ± 0.95, p = 0.05) (Supp. Figs 1g and 2h) and ODN 2216 (3.81 ± 0.98, p = 0.05) (Supp. Figs 1h and 2h) but no other stimuli (Supp. Figs 1i–k and 2h) resulted in significant increase in PI as compared to non-TLR controls (2.72 ± 0.42) (Supp. Figs 1l, and 2h). In non-uveitis TB subjects as well, ODN 2216 (Supp. Fig. 1m) resulted in a significant increase in PI (3.14 ± 0.4) in CD4+ Teff cells as compared to respective non-TLR controls (2.64 ± 0.25) (p = 0.03) (Supp. Figs 1r, and 2j). Other stimuli had no significant effect on CD4+ Teff cell proliferation (Supp. Figs 1n–q and 2j).

Next, we compared the impact of TLR stimulation on proliferation of Teff cells between IOTB, non-uveitis, non-TB uveitis and non-uveitis TB subject groups. To eliminate subject-to-subject variation, we divided PI after TLR stimulation with that of non-TLR controls to normalize the response for each subject. The normalized values were then compared between subject groups. The data obtained showed a significantly lower proliferation after Pam2CSK4 stimulation in subjects with IOTB (0.99 ± 0.07) as compared to non-TB uveitis (1.27 ± 0.08, p = 0.031) subjects and non-uveitis controls (1.29 ± 0.08, p = 0.028) (Fig. 2k). ODN 2216 stimulation also resulted in significantly lower proliferation in IOTB subjects (1.28 ± 0.12) as compared to non-uveitis controls (1.58 ± 0.15, p = 0.05) (Fig. 2l). However, post ODN 2216 stimulation similar proliferation indices were observed in non-uveitis TB and IOTB groups (p = 0.69). Also, TLR4 stimulation showed similar proliferative response between IOTB and non-uveitis (p = 0.72) or non-TB uveitis subjects (p = 1.0) but a higher response than in non-uveitis TB subjects (p = 0.03) (Fig. 2m). These findings collectively corroborated with the data on gene expression patterns in vitreous humor, and showed that the TLR2 and TLR9 mediated CD4+ T cell proliferative responses are diminished in IOTB.

### TLR2 and TLR9 ligation results in lower induction of Tregs in IOTB

Keeping in mind the potential of TLRs to modulate the function of Tregs^16–18^, we assessed the role of TLR ligands in induction of Tregs by comparing the frequency of induced Tregs within and between subject groups.

**Figure 3:**
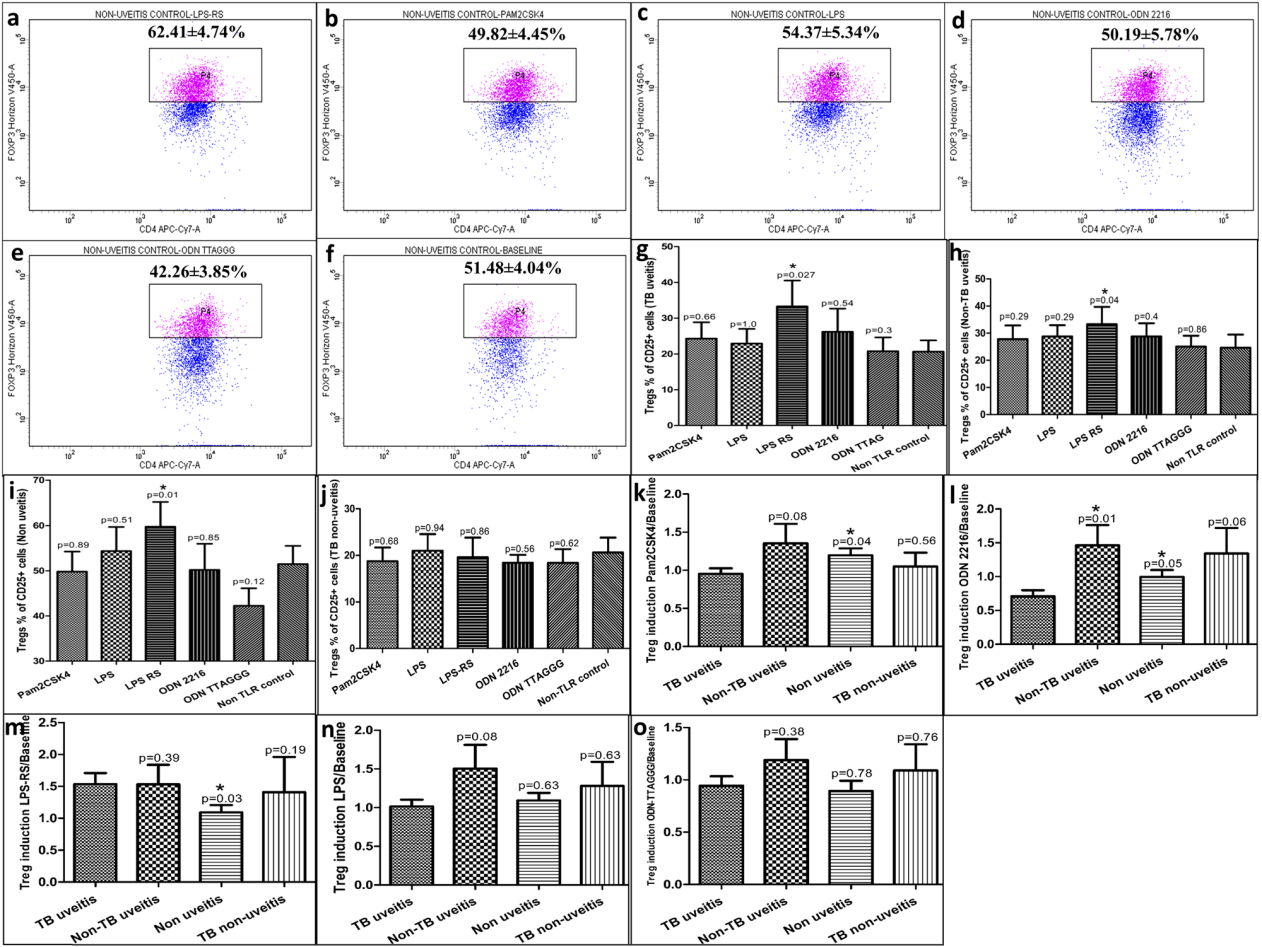
The effect of TLR stimulation on induction of Tregs from CD4+ Teff cells in non-uveitis group. CD4+ Teff cells were treated with ligands of TLR2, TLR4 and TLR9 to assess induction of Tregs by gating FoxP3+ CD4+CD25+ cells from fluorescence minus one tube in all subjects. Representative images from a non-uveitis control subject show the mean frequency of gated Tregs, taking CD4+CD25+ T cells as parent, after stimulation with (**a**) LPS-RS, (**b**) Pam2CSK4, (**c**) LPS, (**d**) ODN 2216, (**e**) ODN TTAGGG and (**f**) non TLR control (baseline) (anti CD3/CD28 + IL-2 only). Treatment with LPS-RS resulted in a significant increase in percentage of Tregs in (**g**) TB uveitis (p = 0.027), (**h**) Non-TB uveitis (p = 0.04) and (**i**) non-uveitis subjects (p = 0.01). (**j**) Differential TLR stimulation resulted in similar percentage of induced Tregs in subjects with non-uveitis TB. (**k**) Pam2CSK4 treatment resulted in significantly lower induction of Tregs in IOTB as compared to non-uveitis controls (p = 0.04). (**l**) ODN 2216 stimulation resulted in significantly lower induction of Tregs in IOTB subjects as compared to non-TB uveitis (p = 0.01) and non-uveitis (p = 0.05) controls, and apparently lower than in non-uveitis TB controls (p = 0.06). Treatment with (**m**) LPS-RS, (**n**) LPS or (**o**) ODN TTAGGG did not cause any decrease in induction of Tregs in subjects with IOTB.

Within group comparison showed that in non-uveitis control subjects, there was a significant increase in frequency of induced Tregs after treatment of Teff cells with LPS-RS (62.41 ± 4.74%, p = 0.014) (Fig. 3a,i) as compared to non-TLR controls (51.48 ± 4.04%) (Fig. 3f,i). However, Pam2CSK4 (p = 0.89) (Fig. 3b,i), LPS (p = 0.51) (Fig. 3c,i), ODN 2216 (p = 0.85) (Fig. 3d,i) or ODN TTAGGG (p = 0.12) (Fig. 3e,i) treatment resulted in similar induction of Tregs as in non-TLR controls. Similar trend was observed following LPS-RS treatment in case of IOTB (33.29 ± 7.23% vs 20.66 ± 3.16%, p = 0.027) (Supp. Figs 2a and 3g) and non-TB uveitis (33.31 ± 6.38% vs 24.64 ± 4.82%, p = 0.04) (Supp. Fig. 2g and 3h) subject groups, with significant increase in frequency of induced Tregs as compared to respective non-TLR controls. Other TLR stimuli resulted in similar induction of Tregs within the IOTB (Supp. Figs 2b–f and 3g) and non-TB uveitis (Supp. Figs 2h–l and 3h) groups. In non-uveitis TB subjects, all TLR stimuli showed similar induction of Tregs as observed in non-TLR controls (Supp Figs 2m–r and 3j).

Overall, in non-TLR controls, frequencies of induced Tregs were lower in IOTB (20.66 ± 3.16%) (Fig. 3g), non-TB uveitis (24.64 ± 4.82%) (Fig. 3h) and non-uveitis TB (20.6 ± 3.22%) (Fig. 3j) as compared to non-uveitis control group (51.48 ± 4.04%) (Fig. 3i) with high intra-group variation. However, frequency of induced Tregs was similar in non-TLR controls between IOTB, non-TB uveitis and non-uveitis TB groups.

Therefore, in order to assess collective differences between IOTB and control groups, we normalized the data by calculating the ratio of frequency of Tregs obtained in TLR stimulated versus non-TLR control cultures for each subject and compared these ratios between the subject groups. Pam2CSK4 stimulation resulted in a lower induction of Tregs in IOTB as compared to non-uveitis (p = 0.04) and apparently lower than non-TB uveitis groups (p = 0.08) but not in non-uveitis TB group (p = 0.56) (Fig. 3k). Similarly, ODN 2216 treatment also resulted in a lower induction of Tregs in IOTB as compared to non-uveitis (p = 0.05), non-TB uveitis controls (p = 0.01) and apparently lower than non-uveitis TB subjects (p = 0.06) (Fig. 3l). Surprisingly, LPS-RS treatment showed significantly higher induction of Tregs in IOTB as compared to non-uveitis (p = 0.03) but not the non-TB uveitis (p = 0.39) and non-uveitis TB groups (p = 0.19) (Fig. 3m). TLR4 stimulation using LPS or stimulation with TLR9 antagonist did not show any significant difference in IOTB as compared to other groups (Fig. 3n,o). Treatment with TLR2 and TLR9 agonists resulted in similar induction of Tregs in non-TB uveitis, non-uveitis and non-uveitis TB groups, highlighting that lower induction of Tregs is specific to IOTB subjects and that induction of Tregs following direct ligation of TLR2 and TLR9 in CD4+ T cells was compromised in IOTB.

### ODN 2216 stimulation results in heightened proinflammatory cytokine response in CD4+ Teff cells in IOTB

We have earlier shown that lower TGF-β expression in Tregs is accompanied with heightened proinflammatory cytokine responses in subjects with IOTB^10^. Keeping this in mind, we assessed the intracellular expression of proinflammatory, IFN-γ and IL-17A and anti-inflammatory, TGF-β and IL-10 cytokines by calculating and comparing the fold change, after stimulation of Teff cells with ligands of TLR2, TLR4 and TLR9.

**Figure 4:**
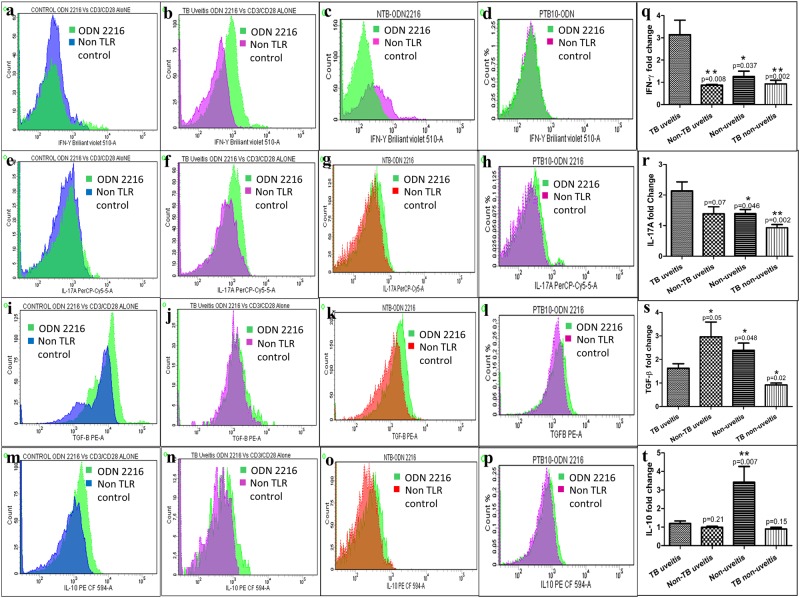
The effect of TLR stimulation on intracellular cytokine expression in CD4+ Teff cells. CD4+ Teff cells were stimulated with ligands of TLRs and the intracellular expression of cytokines was evaluated and compared among subjects. In each group, the relative levels of cytokines were presented as overlay of test treatment over the non-TLR controls. The representative flowcytograms from a non-uveitis control, IOTB, non-TB uveitis and non-uveitis TB subject showing the effect of TLR9 stimulation using ODN 2216 on the intracellular expression of IFN-γ (**a**–**d**), IL-17a (**e**–**h**), TGF-β (**i**–**l**) and IL-10 (**m**–**p**). We observed a significant increase in (**m**) IFN-γ and (**n**) IL-17a expression in IOTB as compared to all other groups, and a significant decrease in (**o**) TGF-β expression, after ODN 2216 stimulation of CD4+ Teff cells. (**p**) The expression of IL-10, after ODN 2216 stimulation was also lower in IOTB subjects.

ODN 2216 stimulation resulted in significantly higher expression of IFN-γ in IOTB (3.14 ± 0.65) as compared to non-uveitis (1.26 ± 0.24, p = 0.037), non-TB uveitis (0.87 ± 0.03, p = 0.008) and non-uveitis TB controls (0.93 ± 0.16, p = 0.002) (Fig. 4a–d,q). Similarly IL-17A expression was also higher in IOTB (2.14 ± 0.29) as compared to non-uveitis control (1.39 ± 0.13, p = 0.046), non-uveitis TB (0.92 ± 0.11, p = 0.002) and apparently higher than in non-TB uveitis group (1.39 ± 0.23, p = 0.07) (Fig. 4e–h,r). However, the expression of anti-inflammatory TGF-β was lower in IOTB (1.63 ± 0.19) than non-uveitis (2.38 ± 0.31, p = 0.048) and non-TB uveitis (2.79 ± 0.65, p = 0.05) and higher than non-uveitis TB groups (0.92 ± 0.08, p = 0.02) (Fig. 4i–l,s). The levels of IL-10 were lower in IOTB (1.89 ± 0.14) as compared to non-uveitis control group (3.42 ± 0.85, p = 0.007) (Fig. 4m–p,t).

The data of cytokine expression by CD4+ Teff cells following stimulation with other TLR ligands including TLR2 (Pam2CSK4) and TLR4 (LPS) in all subject groups is shown in Supplementary Figs 3 and 4, respectively. From these observations, we infer that TLR9 stimulation of CD4+ Teff cells in IOTB results in exaggerated proinflammatory cytokine response, which can contribute to the inflammation observed in the ocular microenvironment.

### TLR9 ligation in Teff cells results in subdued activation of TLR9 signalling pathway in IOTB

**Figure 5:**
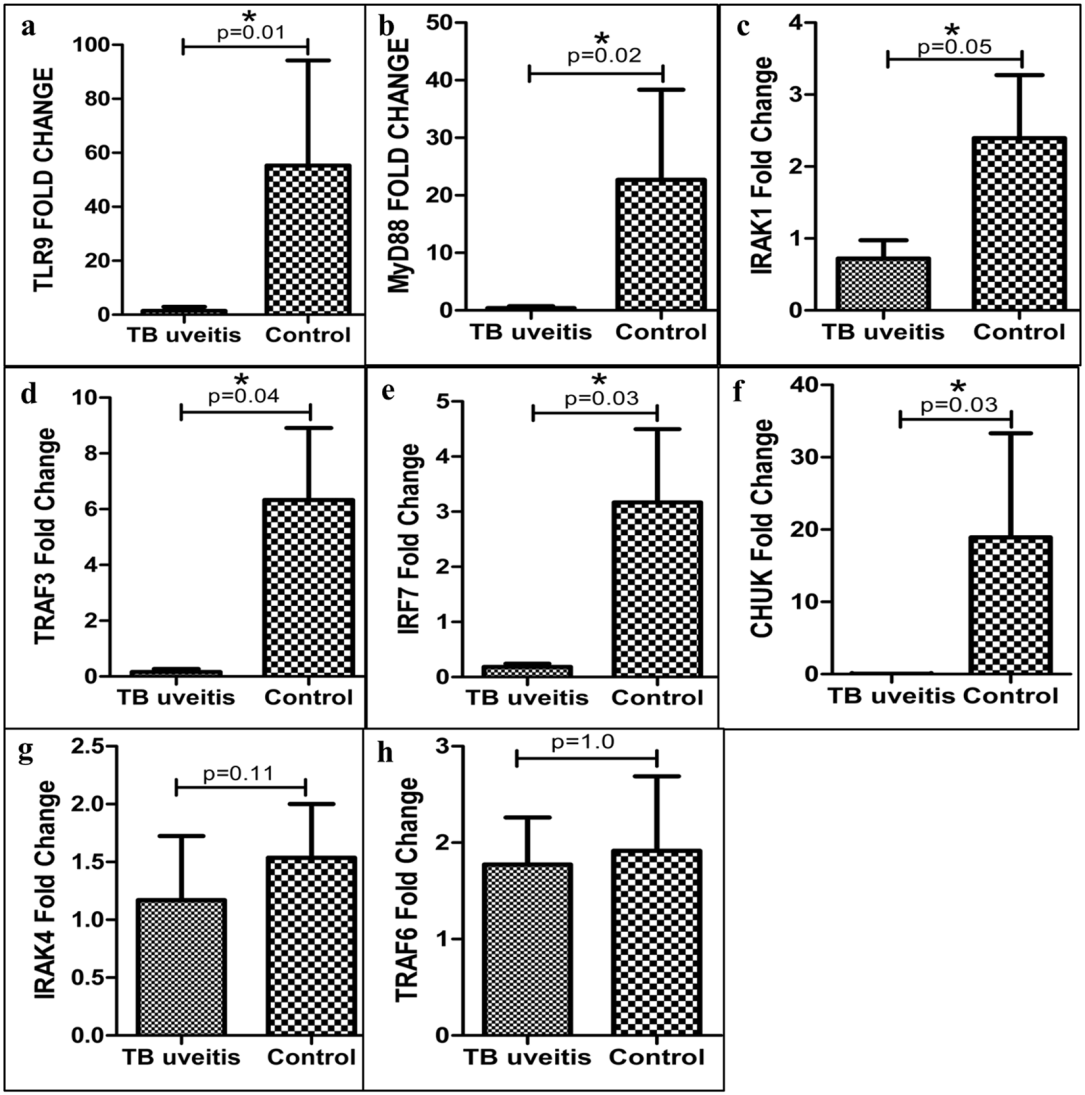
The effect of ODN 2216 stimulation on activation of TLR9 signalling in CD4+ Teff cells. CD4+ Teff cells were stimulated using ODN 2216 for 14 hr and its effect on expression of genes involved in TLR9 signalling was assessed by real time RT-PCR. We observed a significant decrease in mRNA levels of (**a**) TLR9 (p = 0.01), (**b**) MyD88 (p = 0.02), (**c**) IRAK1 (p = 0.05), (**d**) TRAF3 (p = 0.04), (**e**) IRF7 (p = 0.03) and (**f**) CHUK (p = 0.03) in subjects with IOTB as compared to non-uveitis controls. However, few genes of TLR9 signalling pathway including (**g**) IRAK4 (p = 0.11) and (**h**) TRAF6 (p = 1.0) showed no significant differences in expression levels.

Keeping in view the results of intracellular cytokine expression and the fact that mycobacterial DNA is the only detectable entity in vitreous fluid in IOTB, we assessed signalling of its receptor, TLR9 in CD4+ Teff cells by measuring mRNA expression of major mediators of TLR9 signalling pathway after stimulating sorted CD4+ Teff cells with ODN 2216. Surprisingly, even after stimulation, the expression of TLR9 was significantly lower in IOTB group as compared to non-uveitis controls (p = 0.01) (Fig. 5a). On the similar lines, there was a significant decrease in mRNA levels of MyD88 (p = 0.02) (Fig. 5b), IRAK1 (p = 0.05) (Fig. 5c), TRAF3 (p = 0.04) (Fig. 5d), IRF7 (p = 0.04) (Fig. 5e), CHUK (p = 0.03) (Fig. 5f) but not TRAF6 (p = 1.0) (Fig. 5g) and IRAK4 (p = 0.11) (Fig. 5h). The results thus suggest that a subdued TLR9 signalling in CD4+ T cells is associated with an altered cytokine expression and lower induction of Tregs in IOTB.

### TLR9 mediated CD4+ Teff cell responses are associated with ocular inflammation in IOTB

After observing heightened proinflammatory cytokine expression in CD4+ Teff cells stimulated using ODN 2216, we tried to answer a broader question, whether direct TLR ligation mediated CD4+ Teff cell responses correlate with ocular inflammation in IOTB. We earlier found that TLR2 and TLR9 expression is negatively associated with ocular inflammation in IOTB (Fig. 1). We compared various aspects of CD4+ Teff cell function, including Treg induction, cell proliferation and intracellular cytokine expression after TLR2, TLR4 and TLR9 ligation with ocular inflammation scores (evaluated using number of cells in vitreous humor).

**Figure 6:**
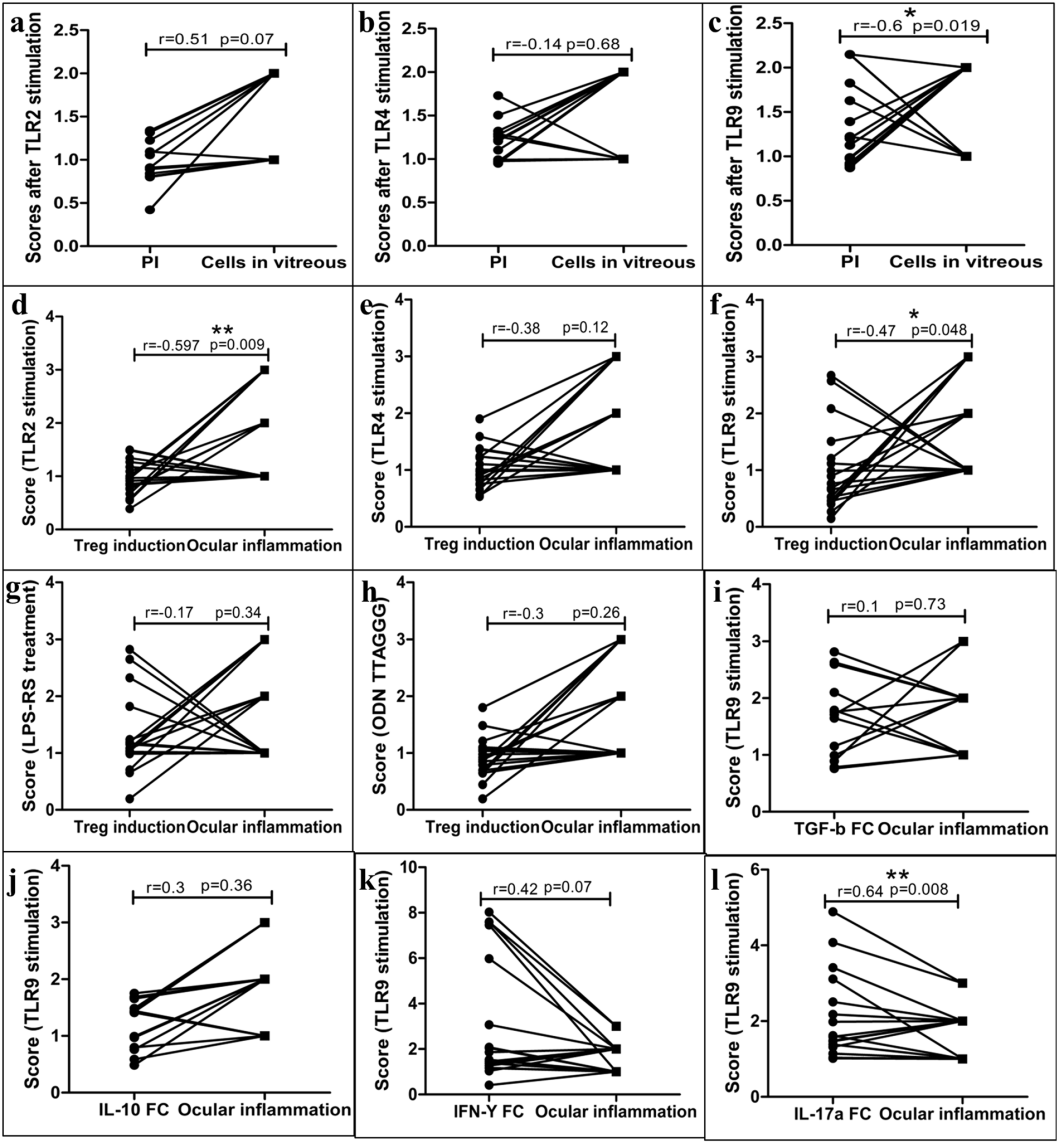
Correlation between TLR mediated CD4+ Teff cell responses and ocular inflammation in IOTB. The correlation between various CD4+ Teff cell function and ocular inflammation was assessed using Pearson correlation test. Ocular inflammation did not show a correlation with Teff cell proliferation after (**a**) TLR2 stimulation by Pam2CSK4 (r = 0.51, p = 0.07) or (**b**) TLR4 stimulation using LPS (r = −0.14, p = 0.68). In contrast, proliferation after TLR9 stimulation using ODN 2216 was negatively associated with ocular inflammation (r = −0.6, p = 0.019). Ocular inflammation observed in IOTB was negatively associated with induction of Tregs after (**d**) TLR2 stimulation (r = −0.597, p = 0.009), but not after (**e**) TLR4 stimulation (r = −0.38, p = 0.12). (**f**) Induction of Tregs after TLR9 stimulation showed a negative correlation with ocular inflammation (r = −0.47, p = 0.048). There was no association between ocular inflammation and induction of Tregs after treatment with (**g**) TLR4 antagonist LPS-RS (r = −0.17, p = 0.34) or (**h**) TLR9 antagonist ODN TTAGGG (r = −0.3, p = 0.26). The comparison of ocular inflammation with cytokine expression in CD4+ Teff cells after TLR9 stimulation showed that ocular inflammation was not associated with expression of anti-inflammatory cytokines including (**i**) TGF-β (r = 0.1, p = 0.73) or (**j**) IL-10 (r = 0.3, p = 0.36). However it showed (**k**) no association with IFN-γ expression (r = 0.42, p = 0.07) but (**l**) a positive association with IL-17A expression (r = 0.64, p = 0.008).

We observed that proliferation of CD4+ Teff cells after TLR2 stimulation using Pam2CSK4 (r = 0.51, p = 0.07) (Fig. 6a) or TLR4 stimulation using LPS (r = −0.14, p = 0.68) (Fig. 6b) was not associated with ocular inflammation in subjects with IOTB. However, proliferation of Teff cells after TLR9 stimulation using ODN 2216 (r = −0.6, p = 0.019) (Fig. 6c) was negatively associated with ocular inflammation in subjects with IOTB.

When we compared induction of Tregs with ocular inflammation, we observed that Treg induction in response to TLR2 (r = −0.597, p = 0.009) (Fig. 6d) and TLR9 (r = −0.47, p = 0.048) (Fig. 6f) stimulation was negatively associated with ocular inflammation, whereas same was not observed after TLR4 stimulation (r = −0.38, p = 0.12) (Fig. 6e). This indicated that the lower induction of Tregs in response to TLR2 and TLR9 stimulation was associated with higher local inflammation in IOTB. In contrast treatment with TLR4 antagonist, LPS-RS (r = −0.17, p = 0.34) (Fig. 6g) or TLR9 antagonist, ODN TTAGGG (r = −0.3, p = 0.26) (Fig. 6h) was not associated with ocular inflammation. Considering the presence of mycobacterial DNA in vitreous fluids of these subjects, we compared the association between ocular inflammation and cytokine response after ligation of TLR9 in CD4+ Teff cells. There was no significant association between ocular inflammation and intracellular levels of TGF-β (r = 0.1, p = 0.73) (Fig. 6i) or IL-10 (r = 0.3, p = 0.36) (Fig. 6j). However, the intracellular levels of IFN-γ showed apparently positive correlation (r = 0.42, p = 0.07) (Fig. 6k), while IL-17A showed positive association with ocular inflammation (r = 0.64, p = 0.008) (Fig. 6l). These results again suggested that subdued TLR9 signalling in CD4+ Teff cells might be responsible for the observed local proinflammatory responses in IOTB subjects.

## Discussion

IOTB is an underdiagnosed uveitic disease, as the proportion of subjects presenting with its clinical signatures is quite low, mostly requiring treatment with anti-tubercular therapy (ATT)^23,24^. Like pulmonary tuberculosis^25,26^, variety of host factors including, genetic makeup, antigenic exposure, initial innate immune responses and the ensuing adaptive immune responses may determine the clinical outcomes in IOTB. Polymorphisms in TLR genes^27–30^, IL-10^26^ and defective TLR9 responses have been reported to increase susceptibility to pulmonary tuberculosis^15^. However, there is lacuna in knowledge about the immune responses in pathogenesis of IOTB. The sudden appearance of ocular inflammation observed in the disease prompted us to understand the role of TLRs in disease pathogenesis.

As key TLRs responding to *Mycobacterium tuberculosis*, we assessed the expression of TLR2, TLR4 and TLR9 in cells of vitreous humor in IOTB. The differences were more striking between IOTB and non-TB uveitis subjects. The reasons for observed lower TLR2 and TLR9 expression in vitreous fluids were not explored in this study, though it gave an initial idea about the resulting local immune responses. Relatively lower expression of TLRs in non-uveitis subjects may be explained by the fact that they lack ocular inflammation in contrast to tubercular and non-TB uveitis subjects. In general, inflammation is expected to be associated with TLR expression. However, in IOTB, mycobacterial factors might be involved in modulating TLR expression on local infiltrating T cells or the previous exposure of these T cells to mycobacterial factors (as most of the IOTB subjects demonstrated latent tuberculosis). This can also be inferred from our recent study showing lower uptake of TLR9 agonist in vitreous humor CD4+ T cells than those in peripheral blood^13^. The decrease in TLR2 and TLR9 expression had a negative correlation with inflammation at local site. These observations also fall in line with previous studies, showing increased susceptibility of TLR2 and TLR9 deficient mice towards pulmonary tuberculosis^14^. Moreover, ATT treatment has been shown to increase the expression of TLR2 and TLR4 in tuberculosis, indicating their association with disease protection^31^. In view of our results and a recent study showing that infiltrating T cells constitute a major proportion of cells in vitreous humor of IOTB^13,32^, the lower expression of TLR2 and TLR9 in ocular infiltrating cells suggests a role for direct TLR engagement on T cells in the disease. In contrast to TLR2 and TLR9, expression of TLR4 and its responses were found to be comparable to controls, suggesting that optimal expression of TLRs is required for a balanced adaptive immune response.

TLR2 ligation has been shown to increase the proliferation of Tregs^17^, while TLR9 agonists can act directly, even in the absence of APCs^20–22,33^. However, CD4+ Teff cells of IOTB subjects showed lower proliferation, in response to ODN 2216 and Pam2CSK4, an indication of diminished response towards TLR9 and TLR2 stimuli. We have recently shown that TLR9 ligand is taken up intracellularly by CD4+ Teff cells in the absence of APCs and activates TLR9 signaling^34^. In a separate study, we have further observed lower TLR9 ligand uptake by CD4+ Teff cells infiltrating vitreous humor of IOTB subjects^13^. Since the cell numbers in vitreous humor are very low, which is one of the major limitations in such studies, we chose to work on CD4+ Teff cells sorted from peripheral blood of same subjects for functional analysis. Additionally, in order to eliminate suppressive influences of Tregs, these cells were separated prior to experiments, to have a homogenous population of CD4+CD25− Teff cells. Here, we observed a lower induction of Tregs in IOTB, in response to TLR2 and TLR9 stimulation, further supporting the observations of a hypo TLR mediated CD4+ Teff cell response. We have previously reported that frequency of Tregs in IOTB is decreased^10^. The failure in induction of Tregs could be one of the contributing factors for the observed decrease in Treg frequency. Considering that mycobacterial DNA is a consistently detectable antigenic entity in IOTB and that CD4+ Teff cells infiltrate vitreous fluid, we chose TLR9 ligand, ODN 2216, to further understand the effects of TLR9 ligation on CD4+ Teff cells. It may be noted that positive impact of direct TLR9 signalling on survival of CD4+ T cells has been described previously in two studies^20,21^. In gene expression analysis, we further observed that major mediators of TLR9 signalling pathway, including, MyD88, IRF7 were expressed at very low levels suggesting that the observed defects in Teff cell responses in IOTB were traceable to TLR9 signalling. Further, by which mechanisms the anti-inflammatory mediators are affected by this subdued TLR9 signalling remains to be seen. ODN stimulation has been previously shown to exert anti-inflammatory responses via increased expression of Indoleamine-pyrrole 2,3-dioxygenase (IDO1), a product of tryptophan metabolism with potent suppressive activity^35^. In this context, we also assessed mRNA levels of IDO1 and NF-kB in CD4+ Teff cells after ODN 2216 stimulation. We observed lower expression of IDO1 (p = 0.050) (Supp. Fig. 5a) and NF-kB (p = 0.052) (Supp. Fig. 5b) in subjects with IOTB as compared to non-uveitis subjects. This indicated that the lower expression of IDO1 and thereby lower function of TLR9-IDO1 axis, post ODN 2216 stimulation possibly contribute to observed lower anti-inflammatory responses in IOTB. This is also significant as IDO1 has been shown to control Th17 responses in human *Mycobacterium tuberculosis* infections^36^. In the event of lower IDO1 expression, proinflammatory responses are expected to dominate and cause ensuing inflammation, as seen in our subjects with IOTB. NF-kB, on the other hand is a common transcription factor for various cytokines and cellular functions, differential expression of its pathway subunits can influence the outcome of the type of cellular function. It has already been demonstrated that the canonical and non-canonical pathway subunits of NF-kB are crucial for the development and function of Tregs^37,38^, which is compromised in IOTB subjects as seen in this work and previously^10^.

Ocular inflammation is a hallmark of all forms of uveitis and one may speculate that this inflammation could be one of contributing factors of diminished CD4+ Teff responses in subjects with IOTB. To specifically address this issue, we performed similar analysis in non-TB uveitis subjects. Despite similar frequency of induced Tregs in non-TLR control cultures in non-TB uveitis group, the observed decrease was specific to IOTB only. The increased levels of proinflammatory cytokines expressed in CD4+ Teff cells after TLR9 stimulation, showed a positive correlation with ocular inflammation. Another inference that can be drawn here is that diminished CD4+ Teff cell response can be one of the causes of exaggerated inflammation, rather than effect. Also, it can be logically inferred that when such hypo-responsive CD4+ T cells would be present at the local site, their inability to mount anti-inflammatory immune responses would exaggerate the ongoing proinflammatory responses. Indeed, we have previously reported higher Th1 and Th17 responses which is concomitant with decrease in Tregs in the IOTB subjects^10^. As expected, we observed higher intracellular expression of proinflammatory cytokines, IFN-γ and IL-17a and lower levels of anti-inflammatory, IL-10 and TGF-β after ODN 2216 stimulation of CD4+ Teff cells in IOTB.

A previous study in animal model has shown induction of lower proinflammatory responses post APC mediated TLR9 activation, contributing towards increased susceptibility to tuberculosis^14^. On the contrary, direct TLR9 ligation on CD4+ Teff cells resulted in a strong proinflammatory outcome in IOTB. It holds relevance in terms of disease pathophysiology as few studies have shown higher levels of T cell chemoattractants in the aqueous humor, predicting a higher T cell infiltration^8,9^. The observed heightened proinflammatory response after TLR9 ligation *in-vitro*, makes a case about the aberrant ability of ocular T cells to respond to direct antigenic challenge i.e. mycobacterial DNA, consequently resulting in the inflammation. However, further studies will be required to assess whether mycobacterial DNA acts as an initial trigger or, it plays a role in perpetuating the ocular inflammation. This is also relevant as the subjects in our study had active disease at the time of enrolment and we lack information about immune activity at the disease onset. Recently, retinal antigen specific cells were shown to infiltrate the retina only after viral infection, despite presentation to T cells in periphery under homeostatic conditions as well^39^. Also autoreactive cells were reported to be more prominent than ESAT6 specific T cells during TB-associated uveitis^32^. In this context we speculate that mycobacterial DNA may provide that initial trigger, leading to ocular inflammation as observed in IOTB. Since TLRs come in picture before the antigen specific T cell responses, a difference in the local expression of TLRs and hyporesponsiveness of T cells infiltrating vitreous chamber towards these stimuli could lead to autoreactive T cell responses. Secondly, the role of APCs in priming the immune responses also could not be established and remains an open question. Nonetheless, the defects in responsiveness of CD4+ T cells towards the key TLRs acting against *Mycobacterium tuberculosis* persisted in these set of patients. Therefore, it appears that along with infection, host factors as well contribute to proinflammatory responses in IOTB.

Unlike primary tuberculosis, where TLRs facilitate Th1 responses to curb the infection^14^, the outcome of immune response observed in IOTB appears to be harmful to the host. These patients lacked any evidence of tubercular manifestation in any other tissue except eye, which can contribute to contrasting outcome in comparison to primary tuberculosis. A typical example of such contrasting outcome of same immune response is formation of granuloma. IOTB involves choroidal tubercles^5^ and subretinal tubercular granuloma^4^ as major manifestations of tubercular involvement in the eye. Despite being a source of containment of the bacterium in primary tuberculosis^40^, the ensuing inflammatory response is detrimental to the eye in IOTB^41,42^. Eye being an immune-privileged site, expresses various soluble factors that not only suppress local inflammatory responses^43^, but are also capable of inducing systemic regulatory responses including generation of Tregs^44^. However, this homeostasis is lost in IOTB, culminating in intraocular infiltration of activated T cells. The diminished response to direct TLR2 and TLR9 stimulation in CD4+ Teff cells in IOTB may be attributed to exposure to mycobacterium at extraocular sites. Although these patients lacked active or did not manifest tuberculosis in extra-ocular tissues, most of these had latent tuberculosis (positivity to mantoux). In this scenario, passive exposure to MTB could influence hypo-responsiveness of these T cells. To address this issue we performed similar experiments in non-uveitis TB subjects. Here, we observed that post TLR9 stimulation, the induction of Tregs was not compromised and there was lesser production of IFN-γ and IL-17A in contrast with IOTB group. These observations further confirmed that the aberrant responses to TLR2 and TLR9 stimulation are characteristic of IOTB. However, TLR2 and TLR9 stimulation showed lower proliferation of CD4+ Teff cells in non-uveitis TB patients, hinting towards the possible role of mycobacterial factors as immune modulators in patients with IOTB as well. However, the identification of these factors is still an open question for better understanding of the disease pathogenesis.

Besides mycobacterial factors, host factors particularly TLRs have a role to play, as we have seen this in the form of selective migration of T cells in vitreous in TB uveitis with hypo TLR2-9 expression at the inflammatory site. Further, our study also demonstrated that ocular inflammation is positively associated with hypo-responsiveness of the peripheral Teff cells to the TLR stimuli in question, suggesting the contribution of host factors in the pathogenesis of IOTB. Overall, our results show that IOTB is associated with a diminished response to TLR2 and in particular TLR9 signalling in CD4+ Teff cells, which manifests into lower Treg induction and elevated proinflammatory responses. Gene polymorphisms in TLR9 have been shown to predispose individuals towards tuberculosis^27–30^, primary ones being those at positions, 1237 (T > C, rs187084) and 1486 (T > C, rs5743836) upstream of promoter region^45,46^. This raises the question whether the observed responses are due to any defect in TLR9 in the study subjects. To address this question, we assessed polymorphisms in TLR9 gene and none of the subjects showed any polymorphism in the sequenced TLR9 gene loci (Supp. Fig. 5c,d). In view of these results, it appears that rather than defect in TLR9 gene, there is an aberrant response to antigenic triggers mediated by TLR9. However, whether the disease involves TB reactivation from latency still needs to be established. Non-uveitis TB is also likely to have an altered immune response, however the observed ocular inflammation in these uveitis patients is an indicative of aberrant local proinflammatory immune response mediated by infiltrating T cells. Since the IOTB patients do not show any active infection, it appears that this altered response to mycobacterial stimuli does have an important role in disease phenotype.

To conclude, our study provided first insights on the innate immune factors, associated with exaggerated intraocular inflammation in IOTB. The study instigates for further research into the less explored area of TLR mediated T cell responses such as, factors causing underexpression of TLRs or diminished responses to TLR stimuli to understand the mechanisms that shape ensuing adaptive responses. The study warrants further research in applications of appropriate TLR ligands as local immune modulators to suppress excessive inflammation, not only in extrapulmonary tubercular diseases, but also various autoimmune and inflammatory diseases where strong inflammatory responses are undesirable.

## Materials and Methods

### Subject recruitment

In the present study, we recruited 18 subjects with confirmed IOTB along with 15 non-uveitis, 15 subjects with non-TB uveitis and 12 subjects without uveitis but active tuberculosis (non-uveitis TB) as control groups from the departments of Ophthalmology and Pulmonary Medicine, Post Graduate Institute of Medical Education and Research (PGIMER) Chandigarh. This study was carried out in accordance with the recommendations of the Institutional ethics committee of PGIMER with written informed consent from all subjects. The study was approved by the Institutional ethics committee of PGIMER. All subjects gave written informed consent in accordance with the Declaration of Helsinki. All patients in IOTB and non-TB uveitis groups had active uveitis at the time of presentation and number of cells in vitreous was scored for subjects with IOTB as a qualitative measure of ocular inflammation (Suppl Table 1).

The inclusion criteria for the IOTB group were:

1. Diagnosis of IOTB by clinical signs^3^,
2. A positive tuberculin skin test (≥10 mm of induration at 48–72 hours) and,
3. A positive triplex PCR (positivity to one or more of MPB64, IS6110 and protein B genes) in the vitreous fluid^7^.

The inclusion criteria for the non-TB uveitis group were:

1. Subjects with fungal endophthalmitis^47^ or viral uveitis^48^ as diagnosed using respective PCR.
2. Subjects with uveitis clinically confirmed with non-tubercular etiology.
3. A negative triplex PCR of the vitreous fluid for *Mycobacterium tuberculosis*^3^

The inclusion criteria for the non-uveitis TB group were:

1. Recent diagnosis of pulmonary tuberculosis (by standard mycobacterial culture) and anti-tubercular therapy (ATT) naive.
2. No present or past evidence of ocular inflammation.

Patients with a history of ATT or those who had undergone any vitreo-retinal surgery in the past were excluded from the study.

### Sample collection and processing

Vitreous fluid was collected from subjects undergoing pars plana vitrectomy (PPV) (IOTB, non-TB uveitis and non-uveitis) and transferred aseptically into micro-centrifuge tubes, followed by centrifugation at 800 × g for 10 minutes. The supernatant was transferred into fresh cryovials and stored at −80 °C for future studies. Vitreous humor cells remaining in the pellet were processed for RNA isolation using RNA aqueous micro kit^TM^ (Thermofisher Scientific, Waltham, MA) and subsequent cDNA synthesis immediately using high capacity cDNA reverse transcription kit (Thermo Fisher Scientific), as per manufacturer instructions. The cDNA samples were used for studying gene expression of TLR2, TLR4 and TLR9. Fifteen ml of peripheral blood was also collected from all subjects included in the study. Peripheral blood mononuclear cells (PBMCs) were purified from the blood samples using lymphoprep^TM^ density gradient centrifugation, as per manufacturer instructions (Stem cell Technologies, Vancouver, Canada) and processed further. PBMC purification, magnetic cell sorting and culture work from all subjects was performed in a cell culture laboratory inside biosafety level 2 containment facility, while following the recommended guidelines^49^.

### Effector T cell sorting

CD4+CD25− T cells (Teff cells) were sorted from PBMCs using human CD4+CD25^hi^ T cell isolation kit (Stem cell technologies). The percent purity of cellular subsets was assessed using anti-human CD3 Alexa Fluor 700 (Clone OKT3), CD4 APC-Cy7 (clone RPA-T4) and CD25 PE-Cy7 (clone BC96) (Biolegend Inc., San Diego, CA) antibodies by flow cytometry (Suppl. Fig. 6). Purity of isolated cells ranged between 90–98%.

### T cell proliferation assay

The isolated Teff cells were labelled with carboxyfluorescein succinimidyl ester (CFSE) (Sigma Aldrich, St. Louis, MI) or efluor 670 cell proliferation dye (Thermofisher Scientific) to assess the effect of TLR ligands on the proliferation of cells. Briefly 10^6^ Teff cells were labelled with 5 μM of CFSE and plated in 48 well plates at final seeding density of 10^5^ cells per well. The cells were stimulated with various TLR ligands in separate sets of experiments (Supp. Table 2) in CTS T cell optimizer^TM^ expansion serum-free medium (SFM) (Thermofisher Scientific) containing Dynabeads® Human T-Activator CD3/CD28 (1 μL/well) (Thermofisher Scientific) and 50 IU recombinant human IL-2 (Peprotech, Rocky Hill, NJ) at 37 °C and 5% CO_2_ for 5 days. The cells stimulated using human T cell activator and IL-2 alone served as non-TLR controls. The data was acquired on FITC channel using FACS diva software, version 7.0 (BD Biosciences, San Jose, CA) on BD LSR Fortessa flow cytometer (BD Biosciences). The proliferation indices (PI) upon stimulation were calculated using the formula described below^50^ and compared between the subject groups.

1 $${\rm{Proliferation}}\,{\rm{index}}=\frac{{\rm{number}}\,{\rm{of}}\,{\rm{cells}}\,{\rm{in}}\,{\rm{all}}\,{\rm{generations}}}{{\rm{number}}\,{\rm{of}}\,{\rm{original}}\,{\rm{parent}}\,{\rm{cells}}}$$

where,

Number of cells in all generations = P1 + P2 + P3 + P4 + P5.

(P1-number of events in the right most peak and so on)

2 $${\rm{Number}}\,{\rm{of}}\,{\rm{original}}\,{\rm{parent}}\,{\rm{cells}}=P1+\frac{P2}{2}+\frac{P3}{4}+\frac{P4}{8}+\frac{P5}{16}+\cdots $$

### Treg induction assay and intracellular cytokine analysis

To assess the effects of direct TLR ligation on induction of Tregs, we performed Treg induction assay on Teff cells using TLR2, TLR4 and TLR9 ligands. Briefly, 10^5^ Teff cells were cultured in presence of TLR ligands (Supp. Table 2) in CTS T cell optimizer expansion SFM containing 100 IU/well recombinant human IL-2 and Dynabeads human T cell activator cocktail, for 3 days at 37 °C, 5% CO_2_. The cells stimulated using human T cell activator and IL-2 alone served as non-TLR controls. In view of intracellular cytokine estimation, protein transport was inhibited by adding monensin (Sigma Aldrich) (2 μM) in cells, 5 hours prior to culture termination. The cultures were terminated and cells were stained for markers of Tregs and intracellular cytokines. Briefly, the harvested cells were surface stained using anti-human CD3 Alexa Fluor 700, CD4 APC-Cy7 and CD25 PE-Cy7 and incubated for 30 minutes at room temperature in dark. The cells were then washed with phosphate buffer saline (PBS) (pH 7.4) and permeabilized using FoxP3 staining buffer (Biolegend Inc.). The permeabilized cells were stained intracellularly for anti-human FoxP3 Horizon V450 (Clone 236 A/E7) (BD Biosciences), CTLA4 APC (clone L3D10) (Biolegend Inc.), IL-10 PE-CF594 (clone JES3-19F1) (BD Biosciences), IL-17A PerCP-Cy5.5 (clone BL168) (Biolegend Inc.), IFN-γ Brilliant Violet 510 (clone 4 S.B3) (Biolegend Inc.) and TGF-β1 PE (clone TB7-16B4) (e-Biosciences, San Diego, CA) followed by incubation for 30 minutes. The cells were washed twice with PBS and acquired on BD LSR Fortessa^TM^ Flowcytometer and data analysed using BD FACS Diva software. Tregs were gated for FoxP3 as the daughter population of CD4+CD25+ T cells and the frequency of Tregs was reported as percent of CD4+CD25+ T cells. The frequency of induced Tregs was compared between subject groups. In order to overcome subject variation, the percentage induction was normalized by dividing the frequency of induced Tregs in presence of TLR stimulation with that in non-TLR control. The expression of various cytokines on per cell basis was also compared by calculating fold change with respect to non-TLR control for every subject.

### Gene expression

The gene expression of TLR2, TLR4 and TLR9 was assessed using SYBR green based real time RT-PCR (Step one plus ABI Inc. Foster city, CA). Human GAPDH was used as housekeeping control for all gene expression analysis. The primers for all genes were designed using primer blast software (NCBI) (Supp. Table 3). Briefly, the PCR conditions included 40 cycles of denaturation at 95 °C for 30 seconds, annealing at 60 °C for 1 minute, followed by extension at 72 °C for 30 seconds. All real time PCR samples were loaded in triplicates and the mean C_t_ value was used for calculation of fold change using ΔΔC_t_ method.

### Real Time RT-PCR array

The mRNA levels of TLR9 signalling genes including TLR9, MyD88, IRAK1, IRAK4, TRAF3, TRAF6, IRF7 and CHUK were assessed by real time RT-PCR profiler array (Qiagen, Hilden, Germany). Briefly, total RNA was isolated from TLR9 stimulated T cells using Tri reagent (Life technologies, Carlsbad, CA) and processed for cDNA synthesis using RT^2^ first strand kit (Qiagen). TLR9 signalling array was custom synthesized (Supp. Table 4) and 5 ng/well cDNA was used for analysis of mRNA expression. The results of gene expression array were confirmed using real time RT-PCR and the fold changes were calculated and compared using ΔΔC_t_ method. The expression of NF-kB and IDO1 was also assessed in samples (n = 8, each group) by real time RT-PCR as described previously^51^.

### TLR9 polymorphism screening

DNA was isolated from peripheral blood of subjects with IOTB, using DNA isolation kit as per manufacturer instructions (Qiagen). Primers for detection of single nucleotide polymorphisms (SNP) were designed and synthesized (Eurofins Scientific Luxembourg). Human TLR9 gene region for detection of SNPs was amplified using conventional PCR and products purified after running agarose gel electrophoresis using PCR clean-up gel extraction kit (Macherey Nagel, Fisher Scientific). The purified products were then sequenced using Sanger sequencing (Hitachi, ABI Inc). The sequences obtained were then aligned with human TLR9 gene and checked for SNPs, particularly at positions 1237 (T > C, rs187084) and 1486 (T > C, rs5743836) upstream of promoter, using nucleotide BLAST (NCBI).

### Statistical Analysis

The data was presented as mean (±SEM) and checked for normality using D’Agostino and Pearson omnibus normality test. Nonparametric data was compared using Mann Whitney test, while normal data was compared using Student’s *t* test. The mRNA levels of TLRs in vitreous humor were compared using Mann Whitney test. The correlation between fold changes of various TLRs and between TLR expression, CD4+ Teff cell responses and ocular inflammation scores was tested using Spearman correlation test. The effect of TLR stimulation on proliferation of Teff cells within non-uveitis control group was compared using Wilcoxon matched-pairs signed rank test while that in IOTB using paired *t* test. The effect of TLR stimulation on Treg induction, intracellular cytokine expression and mRNA levels of TLR9 signalling pathway genes, between the subject groups were compared using unpaired *t* test or Mann Whitney test, depending on data distribution. All statistical analysis was performed using Graphpad prism software version 5.03.

## Electronic supplementary material

Supplementary Data

## Data Availability

All data generated or analysed during this study are included in this published article (and its Supplementary Information files).
