# Supplementary material for: Diminished TLR2-TLR9 mediated CD4+ T cell responses are associated with increased inflammation in intraocular tuberculosis
Source: Sci Rep. 2018 Sep 14;8:13812. doi: 10.1038/s41598-018-32234-3 (PMC6138653; doi:10.1038/s41598-018-32234-3)
Supplement: Supplementary file 1 — Supplementary Data [file 41598_2018_32234_MOESM1_ESM.docx]

**Diminished TLR2-TLR9 mediated CD4+ T cell responses are associated with increased inflammation in intraocular tuberculosis**

Ravi Kumar Sharma, M.Sc^1,2^, Jyoti Sharma, M.Sc ^1^, Zafar K. Khan, Ph.D^2^, Ajinkya Pattekar, M.S^2^, Vishali Gupta, M.S^1^, Reema Bansal, M.S^1^, Kusum Sharma, M.D^3^, Ashutosh Nath Aggarwal, M.D^4^, Amod Gupta, M.S^1^ & Naresh Sachdeva, Ph.D^5^

^1^Advanced Eye Centre, Post Graduate Institute of Medical Education and Research, Chandigarh, India.

^2^Department of Microbiology and Immunology and the Institute for Molecular Medicine and Infectious Disease, Drexel University College of Medicine, Philadelphia, PA, USA.

^3^Department of Medical Microbiology, Post Graduate Institute of Medical Education and Research, Chandigarh, India.

^4^Department of Pulmonary Medicine, Post Graduate Institute of Medical Education and Research, Chandigarh, India.

^5^Department of Endocrinology, Post Graduate Institute of Medical Education and Research, Chandigarh, India.

Correspondence: [naresh_pgi@hotmail.com](mailto:naresh_pgi@hotmail.com)

**SUPPL. TABLE 1: Ocular activity in intraocular tuberculosis group (Ocular inflammation scores)**

| Subject ID | Number of cells in vitreous |
| --- | --- |
| IOTB1 | 2 |
| IOTB2 | 2 |
| IOTB3 | 2 |
| IOTB4 | 2 |
| IOTB5 | 2 |
| IOTB6 | 2 |
| IOTB7 | 2 |
| IOTB8 | 1 |
| IOTB9 | 2 |
| IOTB10 | 2 |
| IOTB11 | 3 |
| IOTB12 | 2 |
| IOTB13 | 2 |
| IOTB14 | 2 |
| IOTB15 | 2 |
| IOTB16 | 2 |
| IOTB17 | 2 |
| IOTB18 | 2 |

**SUPPL. TABLE 2:- Ligands for various toll like receptors (TLRs) used in the study**

| **S. No** | **Ligand** | **Function** | **Working Concentration** |
| --- | --- | --- | --- |
| 1 | Pam2CSK4 | TLR2 agonist | 50 ng/ml |
| 2 | LPS | TLR4 agonist | 1000 ng/ml |
| 3 | LPS-RS | TLR4 antagonist | 2000 ng/ml |
| 4 | ODN 2216 FITC/ODN 2216 | TLR9 agonist | 500 ng/ml |
| 5 | ODN TTAGGG (A51) | TLR9 antagonist | 500 ng/ml |

**SUPPL. TABLE 3:- List of genes analyzed in real time RT-PCR array**

|  |  | **1** |  | **2** |  | **3** | **4** |
| --- | --- | --- | --- | --- | --- | --- | --- |
| **A** |  | TLR9 |  | CHUK (IKK-α) |  | IRF3 | ACTB |
| **B** |  | MYD88 |  | IKBKB |  | IRF7 | RTC |
| **C** |  | IRAK4 |  | IKBKE |  | TBK1 | PPC |
| **D** |  | IRAK1 |  | TAB1 |  | NFKB1 | GDC |
| **E** |  | TRAF6 |  | TAB2 |  | RELA |  |
| **F** |  | UBE2N |  | MAP2K4 |  | CREB1 |  |
| **G** |  | TRAF3 |  | MAP2K7 |  | TANK |  |
| **H** |  | NR2C2 |  | MAPK8IP3 |  | GAPDH |  |

**SUPPL. TABLE 4:- Sequences of primers used for confirmation of RT PCR-array results**

| **S.No** | **Gene** | **Forward Primer 5’-3’** | **Reverse Primer 5’-3’** |
| --- | --- | --- | --- |
| 1. | TLR2 | GGGTCATCA TCAGCCTCTCC | AGGTCACTGTTGCTAATGTAGGTG |
| 2. | TLR4 | CAGAGTTGCTTTCAATGGCATC | AGACTGTAATCAAGAACCTGGAGG |
| 3. | TLR9 | CCACCCTGGAAGAGCTAAACC | GCCGTCCATGAATAGGAAGC |
| 4. | MyD88 | GAGCGTTTCGATGCCTTCAT | GTTTGTCTGTTCCAGTTGCCG |
| 5. | IRAK1 | CGAGGAGTACATCAAGACGGG | TGTGCTCTGGGTGCTTCTCA |
| 6. | IRAK4 | TAGTTCGGCTGGTTCTTCTGTC | CATTGAGGCAGCGCACATA |
| 7. | TRAF3 | GCTGTCCTGACAGAAGAGAAC | TCAGGGACAAAAACTGGCGT |
| 8. | TRAF6 | TCTGTGTCCGTCCTCTACCA | GAGCACACAAAGAAAGCTGGG |
| 9. | IRF7 | CGCCACTGTTTAGGTTTCGC | GCTGCCTCGGTATGGATCTC |
| 10. | GAPDH | GGTGTGAACCATGAGAAGTATGA | GAGTCCTTCCACGATACCAAAG |

**SUPPLEMENTARY FIGURES:**

**
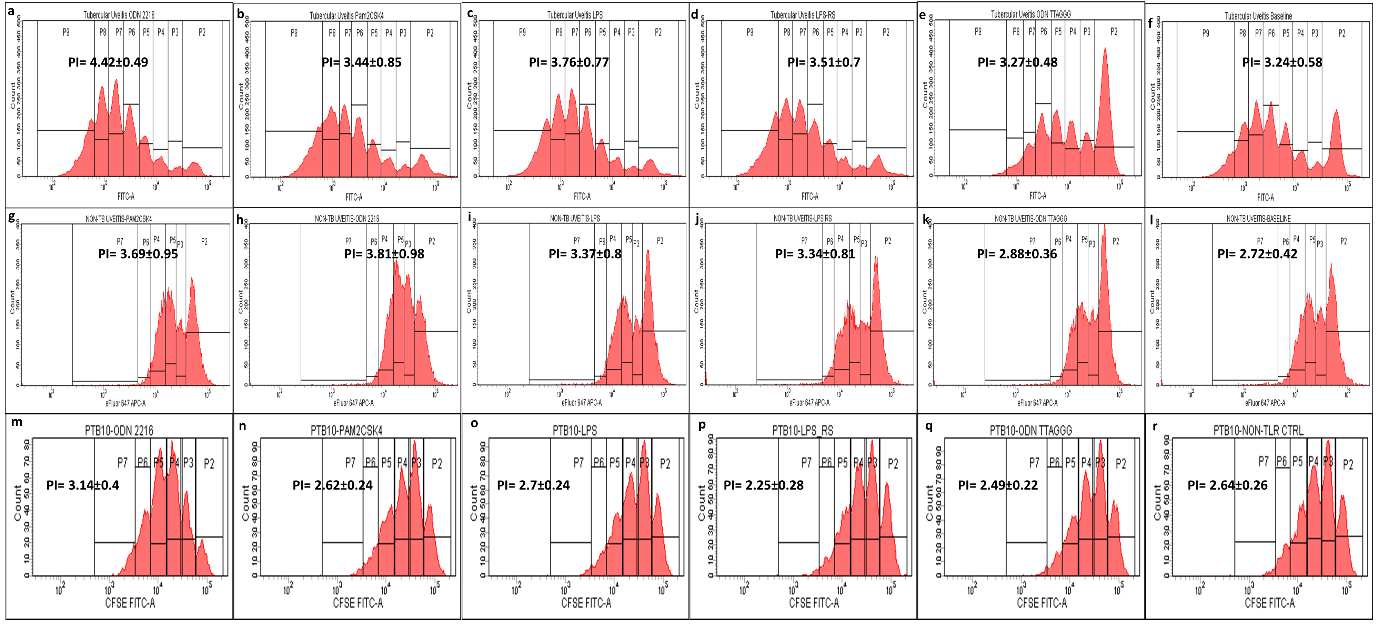
**

**Suppl. Fig. 1: Proliferation of CD4+ Teff cells in response to TLR stimulation:**

CD4+ Teff cells were treated with ligands of TLR2, TLR4 and TLR9 and the effect on proliferation was compared within as well as between subject groups. Representative flowcytograms of an IOTB subject showing proliferation peaks after stimulation with (a) ODN 2216, (b) Pam2CSK4, (c) LPS, (d) LPS-RS, (e) ODN TTAGGG or (f) human T-activator CD3/CD28 alone. Similarly, flowcytograms from a non-TB uveitis show proliferation after stimulation with (g) Pam2CSK4, (h) ODN 2216, (i) LPS, (j) LPS-RS, (k) ODN TTAGGG or (l) human T-activator CD3/CD28 alone. (m-r) Panel shows proliferation peaks in a non-uveitis TB subject after treatment with ODN 2216, Pam2CSK4, LPS, LPS-RS, ODN TTAGGG and human T-activator CD3/CD28 alone, respectively.


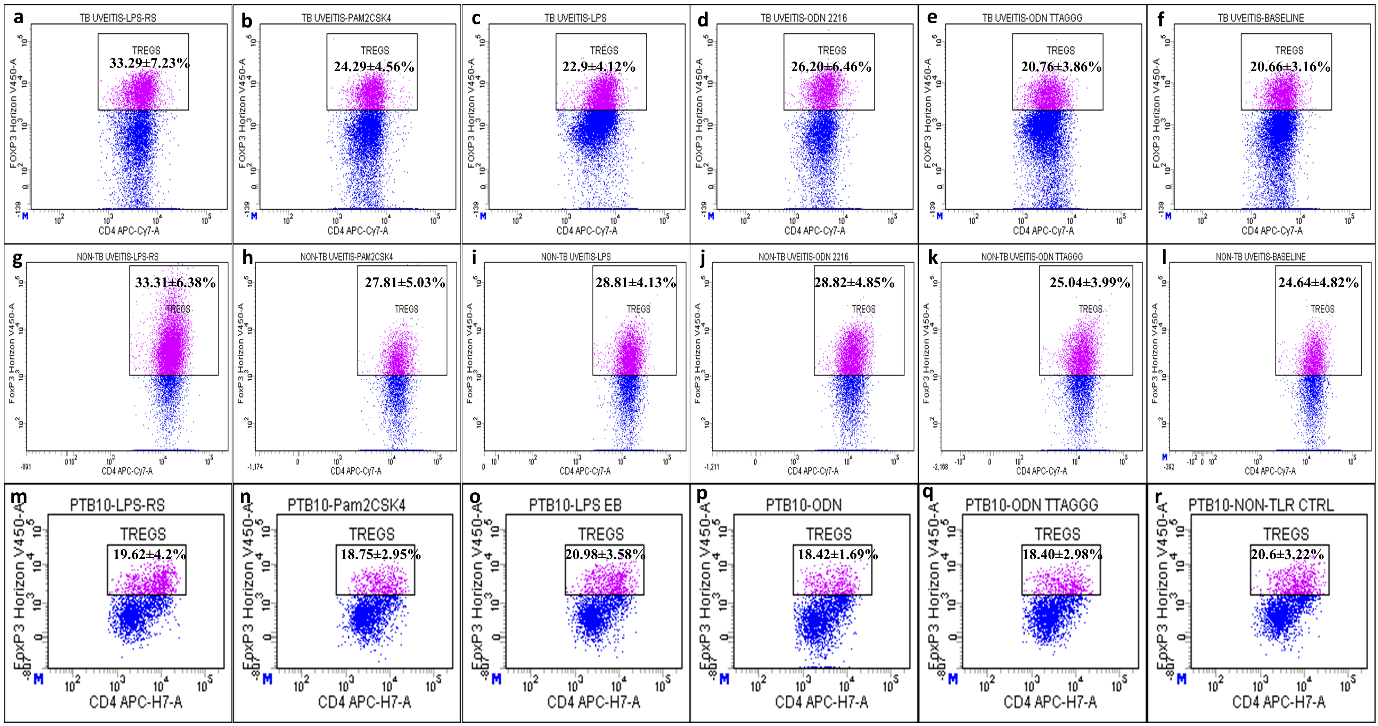


**Suppl. Fig. 2: The effect of TLR ligand stimulation on induction of Tregs:**

Induction of Tregs was also assessed in all subject groups. Representative flowcytograms showing frequency of induced Tregs in IOTB, after treatment with (a) LPS-RS, (b) Pam2CSK4, (c) LPS, (d) ODN 2216, (e) ODN TTAGGG and (f) non-TLR control. Similarly panel (g-l) and (m-r) show representative flowcytograms in non-TB uveitis and non-uveitis TB groups respectively.

**
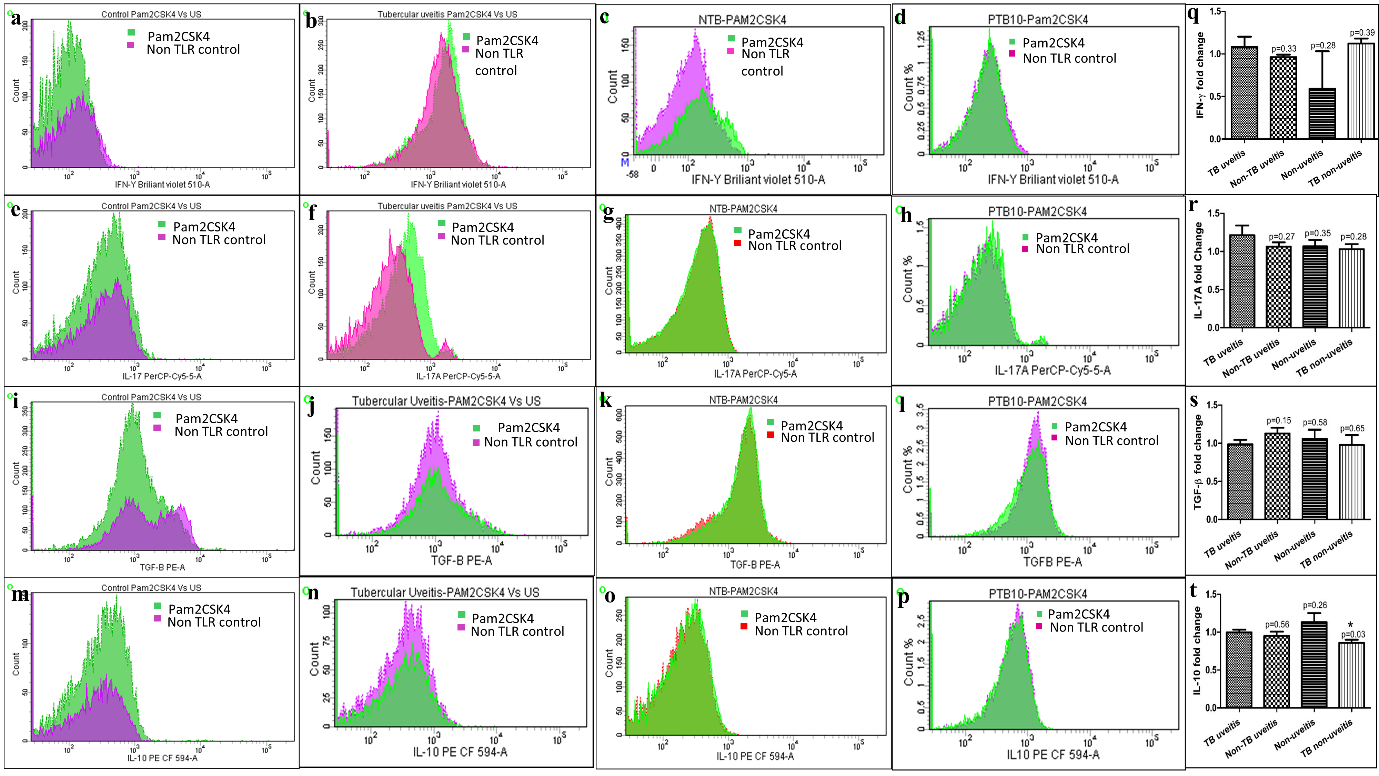
**

**Suppl. Fig. 3:** **Effect of TLR2 stimulation of CD4+Teff cells on cytokine expression:**

The representative flowcytograms from non-uveitis, IOTB, non-TB uveitis and non-uveitis TB subjects, respectively, showing the effect of TLR2 stimulation using Pam2CSK4 on the intracellular expression of (a-d) IFN-γ, (e-h) IL-17A, (i-l) TGF-β and (m-p) IL-10 (q-t).

**
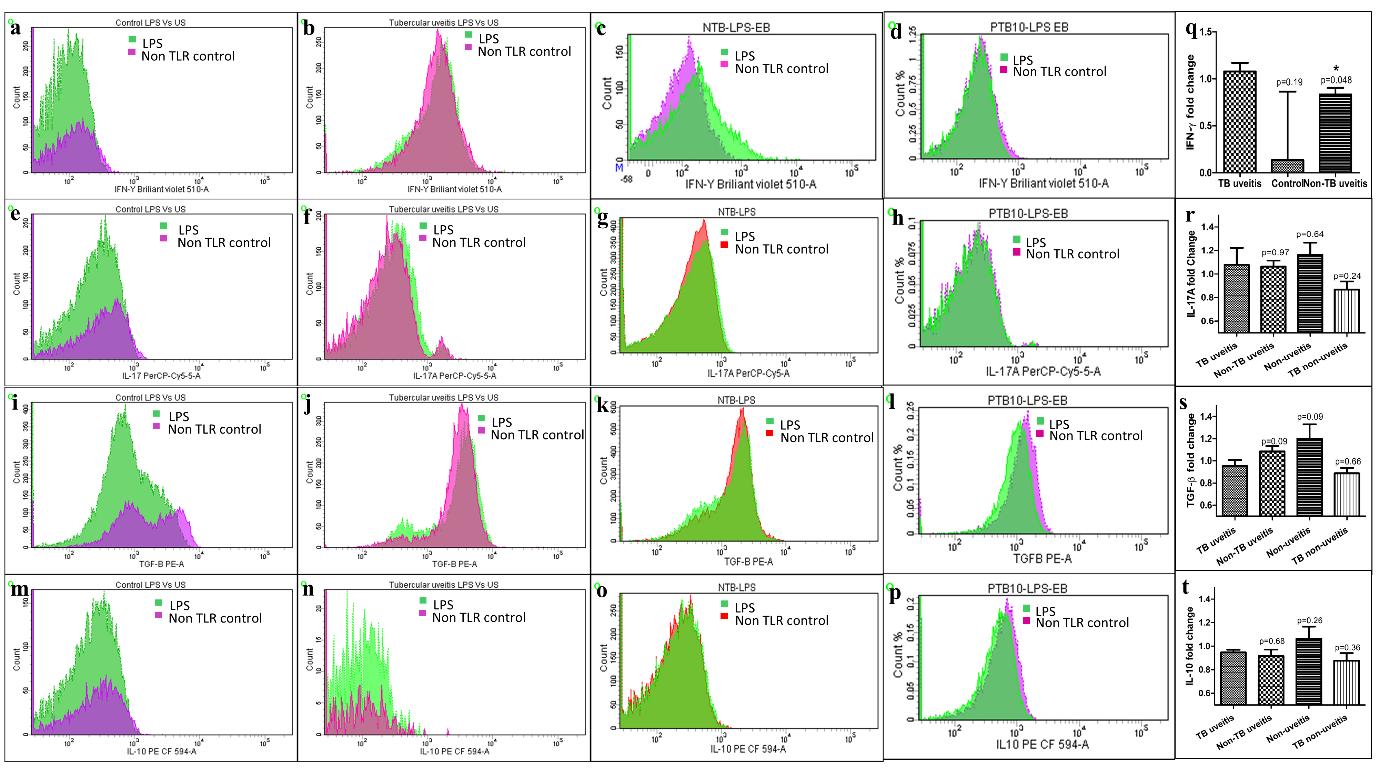
**

**Suppl. Fig. 4:** **Effect of TLR4 stimulation of CD4+Teff cells on cytokine expression:**

The representative flowcytograms from non-uveitis control, IOTB, non-TB uveitis and non-uveitis TB subjects, respectively, showing the effect of TLR4 stimulation using LPS on the intracellular expression of (a-d) IFN-γ, (e-h) IL-17a, (i-l) TGF-β and (m-p) IL-10 (q-t). The intracellular levels of all cytokines were mostly similar across all the groups after TLR4 stimulation.

**
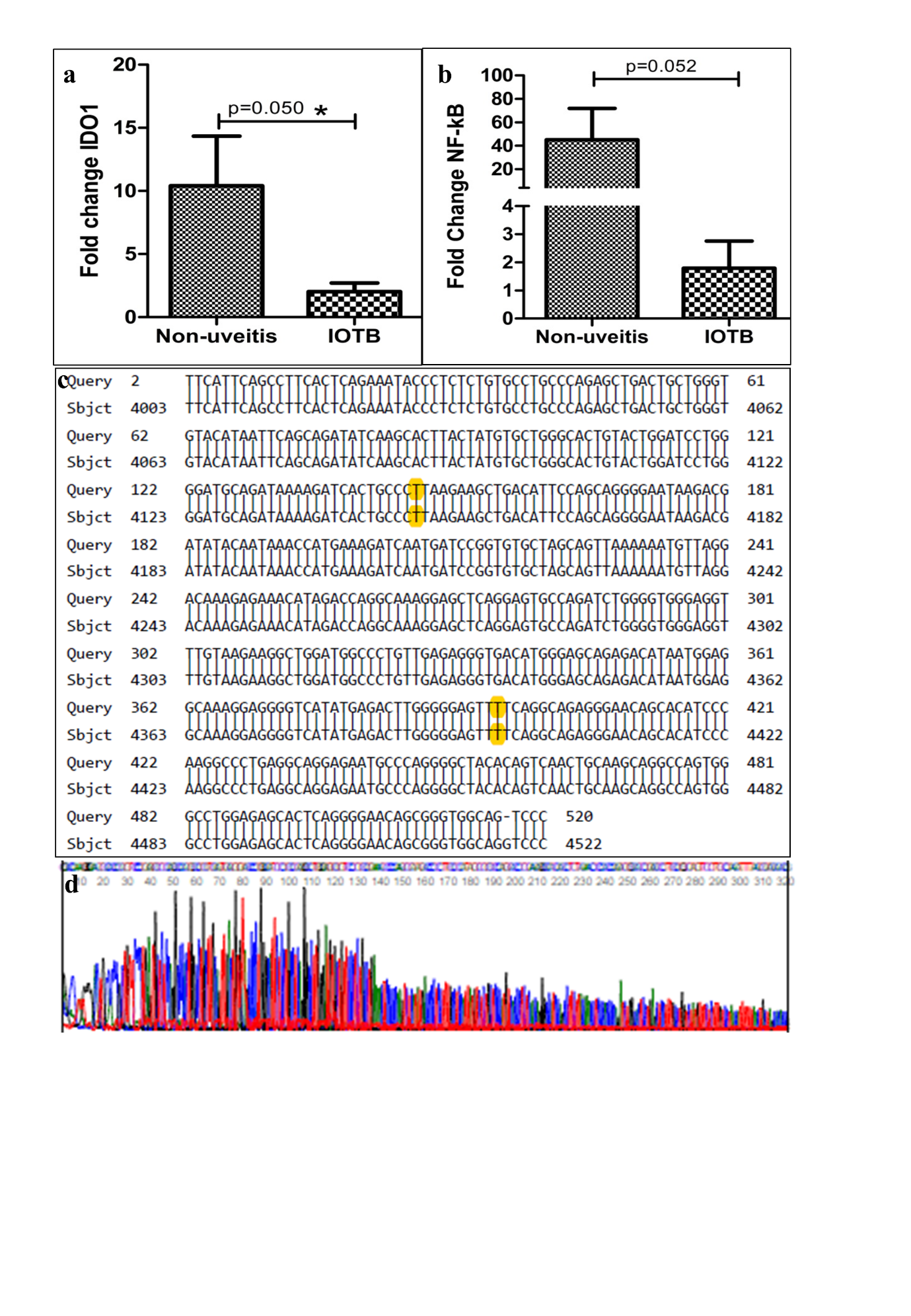
**

**Suppl. Fig. 5: Mechanistic basis of observed responses in IOTB:**

The mRNA expression of IDO1 and NF-kB was assessed after TLR9 stimulation using real time RT-PCR. The expression of (a) IDO-1 (p=0.050) and (b) NF-kB (p=0.052) was observed to be lower in IOTB subjects as compared to non-uveitis subjects. TLR9 single nucleotide polymorphisms (SNP) were assessed using sequencing of PCR products. (c,d) None of the recruited subjects showed SNP in the amplified TLR gene loci including the positions 1237 (T>C, rs187084) or 1486 (T>C, rs5743836) upstream of promoter. The highlighted bases in a representative subject show the location of sequence variants rs187084 and rs5743836 respectively. Figure d shows chromatogram of PCR product sequenced in a representative IOTB subject.

**
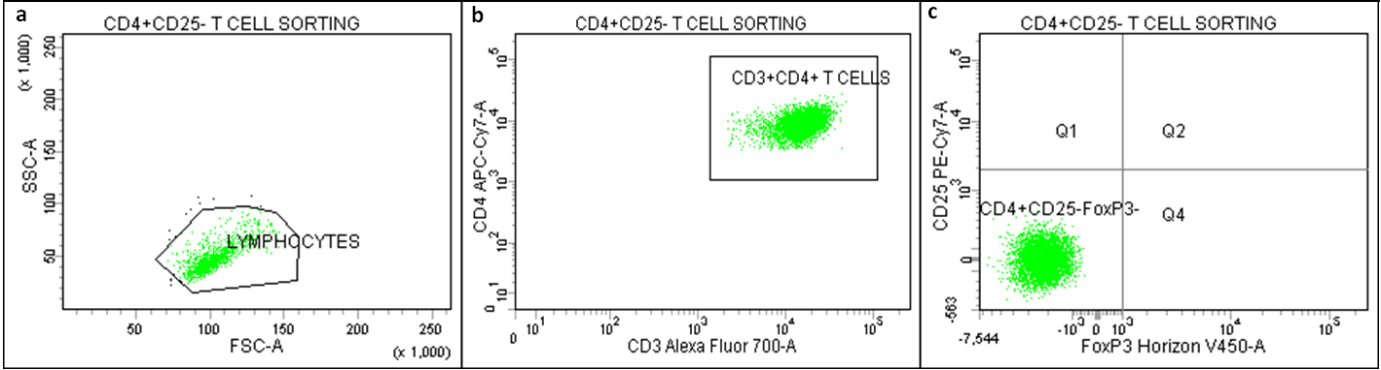
**

**Suppl. Fig. 6: Sorting and assessment of purity of CD4+ Teff cells:**

CD4+CD25-FoxP3- cells were sorted from PBMCs using CD4+CD25^hi^ T cell isolation kit. Sorted CD4+CD25- T cells were used for purity assessment of the sorted populations. (a) Lymphocytes were gated on the basis of forward and side scatter. (b) CD3+CD4+ T cells gated as double positive cells, constituted 90-98% of sorted cells. (c) The sorted cells did not express FoxP3 and CD25, confirming the effector phenotype of these cells.
